# Targeting of Chk2 as a countermeasure to dose-limiting toxicity triggered by topoisomerase-II (TOP2) poisons

**DOI:** 10.18632/oncotarget.8790

**Published:** 2016-04-18

**Authors:** Prashanth Gokare, Arunasalam Navaraj, Shengliang Zhang, Noboru Motoyama, Shen-Shu Sung, Niklas K. Finnberg

**Affiliations:** ^1^ Laboratory of Translational Oncology and Experimental Cancer Therapeutics, Department of Medical Oncology and Molecular Therapeutics Program, Fox Chase Cancer Center, Philadelphia, PA 19111, USA; ^2^ Penn State Hershey Cancer Institute, Penn State Hershey Medical Center, Hershey, PA 17104, USA; ^3^ Institute of Longevity, Department of Cognitive Brain Sciences Research Institute, National Center for Geriatrics and Gerontology, Aichi 474-8511, Japan; ^4^ Department of Pharmacology, Penn State College of Medicine, Hershey, PA 17104, USA

**Keywords:** Chk2, topoisomerase inhibitors, apoptosis, etoposide, myelosuppression

## Abstract

The DNA damage response (DDR) gene cell cycle checkpoint kinase 2 (Chk2) triggers programmed cell death and lethal radiation-induced toxicity in mice *in vivo*. However, it is not well established to what extent targeting of Chk2 may protect from dose-limiting toxicities (DLT) inflicted by mainstay cancer chemotherapy. We screened different classes of chemotherapy in wild type and Chk*2*-deficient cells. Here we show that loss of Chk2 protect from cell death *in vitro* and lethal toxicity *in vivo* following treatment with topoisomerase II (TOP2)–inhibitors whereas no such protection was observed following treatment with topoisomerase I (TOP1) inhibitors. Furthermore, through combined in silico and functional screens of the Diversity Set II (NCI/NTP) chemical library we identified the carbanilide-derivative NSC105171, also known as ptu-23, as a novel Chk2 inhibitor (Chk2i). Indeed, NSC105171 can be administered safely to mice to countermeasure etoposide-induced toxicity. Incorporation of Chk2i into chemotherapy protocols employing TOP2-inhibitors may be an effective strategy to prevent DLT's without interfering with treatment.

## INTRODUCTION

Myelosuppression is one of the most common acute dose-limiting toxicity (DLT) following systemic chemotherapy that may result in delayed or discontinued treatment with a negative impact on patient quality-of-life and survival. As such, there is a need for clinical translation of targeted toxicity countermeasures that prevent killing of normal cells without interfering with the efficacy of treatment. The cell cycle checkpoint kinase 2 (Chk2) is a serine/threonine kinase that transduces DNA damage response (DDR) signals from the kinases ATM and to some extent also ATR. ATM phosphorylates Chk2, promotes dimerization and subsequent trans-autophosphorylation of Serine 516 required for fulminant activity [1, 2]. Loss of the Chk2-gene in mice is associated with reduced levels of apoptosis in hematopoietic cells and improved survival following lethal doses of γ-radiation (IR) [3].

Interestingly, evidence suggests different emphasis on Chk2's function in normal and cancer cells. Chk2 promotes programmed cell death in normal cells in part through the tumor suppressive p53-pathway. In contrast, cancer cells frequently carry inactivating mutations of the p53 gene and increasingly rely on p53-independent cell cycle checkpoints in G2/M something that may be exploited therapeutically [4, 5]. Indeed, Chk2 have been shown to trigger p53-independent but CDC25-dependent cell cycle checkpoints and subsequent DNA repair following damage inflicted by cancer therapy [6]. Consequently, pharmacologic Chk2-inhibitors (Chk2i) have been developed with the intent to sensitize cancer cells to radiochemotherapy. Chk2i's may also at the same time protect normal human and mouse cells from cell death following ionizing radiation *in vitro* [7–9]. However, it remains unclear to what extent pharmacologic Chk2i's are an effective strategy to prevent toxicities from radiochemotherapy *in vivo*.

The use of Chk2i may be particularly beneficial for patients subjected to chemotherapy given that such treatments generate a systemic exposure to genotoxicity that typically contrasts that of modern radiotherapy. However, chemotherapeutics constitutes a heterogeneous group of compounds with diverse modes of action that may not rely entirely on ATM-Chk2-p53 signaling (or DDR at all) to trigger DLT's [10]. It is therefore possible that successful molecular targeting of Chk2 for the purpose of inhibiting chemotherapy DLT is highly treatment-dependent. Successful clinical translation of Chk2i may thus depend on identifying specific context where Chk2 inhibition would be most beneficial as toxicity countermeasure.

To address this, we performed screening in cells that were proficient and deficient for Chk2 (Chk2-/−) in order to identify chemotherapeutics that trigger toxicity through Chk2. Collectively, our data indicates that chemotherapy belonging to the class of topoisomerase II (TOP2) inhibitors is particularly likely to trigger Chk2-dependent cell death and toxicity *in vitro*. Furthermore, we also designed an *in silico* screen that would allow for the condensation of small molecule compound libraries to lead compounds with an affinity to bind to the ADP binding pocket of Chk2. By assessing the Chk2 kinase- and cell death inhibitory activities of the compounds in this condensed library we were able to identify the antiviral compound ptu-23/NSC105171 as a Chk2i that reduces etoposide toxicity *in vivo*.

## RESULTS

### Chk2 triggers cell death in normal tissues following DNA damage inflicted by whole-body gamma-radiation (WBR)

**Figure 1:**
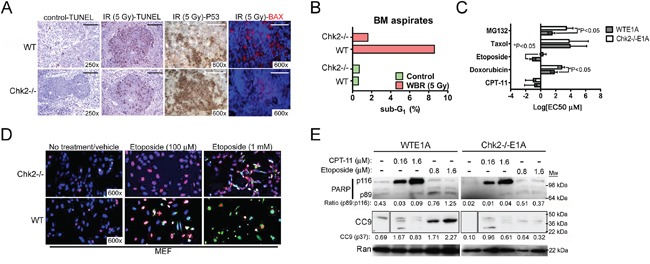
Chk2-targeting protects from toxicity triggered by DNA damage *in vivo* and *in vitro* **A.** Immunohistochemistry for TUNEL (apoptosis), p53 and bax in spleens isolated from wild type and Chk2-/− mice that were subjected to 5 Gy of whole-body ionizing radiation (IR). Representative images are shown. Size bar is 40 μM. **B.** Flow cytometric analysis of the frequency of apoptotic cells with sub-G_1_ DNA present in BM aspirates from the femur and tibia of wild type and Chk2-/− mice subjected to either sham (control) or 5 Gy of whole-body radiation (WBR). **C.** The sub-G_1_ EC_50_ was determined by flow cytometric analysis of E1A-immortalized wild type (WTE1A) and Chk2-/− (Chk2-/−E1A) MEF's following twenty-four (24) hours of treatment with different chemotherapy. Error bars represent the standard deviation from the mean and N=3/treatment. *Indicates P<0.05, Student's t-test. **D.** Immunocytochemistry analysis to detect the presence of p53 (Cy3; red) and cleaved caspase-8 (Cy2; green) in E1A-immortalized wild type (WT) and Chk2-deficient (Chk2-/−) MEF's subjected to treatment with two different doses of etoposide (100 μM and 1 mM). DAPI (blue) was employed to visualize cellular nuclei. Representative images are shown. **E.** Western blot analysis for the detection of full-length PARP (p116), cleaved PARP (p89) and cleaved caspase-9 (CC9; p37) in WTE1A and Chk2-/−E1A MEF's subjected to treatment with CPT-11 and etoposide. Ran was used as a loading control. Densiometry was performed using the NIH Image J 1.45S software. The p89/p116 ratio indicates the densiometric ratio of cleaved PARP p89 to full-length PARP p116 in each lane respectively. Densiometric quantitation of CC9 p37 normalized to the loading control (Ran) are shown.

Previous findings have shown that Chk2 is a mediator of cell death in normal hematopoietic tissues following WBR [11]. Indeed mice proficient (wild type [WT]) and lacking Chk2 (Chk2-/−) were subjected to WBR (5 Gy) and assessed for cell death in hematopoietic organs (Figure 1A and 1B). Consistent with previous data, mice lacking Chk2 showed a reduced frequency of TUNEL-positive (+) cells in their white pulps following radiation exposure compared to littermates indicating ameliorated apoptosis following DNA damage. Furthermore, Chk2-deficient mice were also able to tolerate increased expressions of p53 while at the same time expressing reduced levels of the pro-apoptotic protein Bax in the white pulp as determined by IHC (Figure 1A). This is consistent with prior investigations where Chk2 does not per se appear to be required for the stabilization of p53 protein following DNA damage. However, the induction of downstream target genes required for p53-dependent cell cycle arrest and cell death is hampered by loss of Chk2 [11]. One p53-dependent gene that is regulated positively by Chk2 is MDM2. MDM2 is an E3 ubiquitin-protein ligase that promotes p53 degradation suggesting that p53 protein half-life may potentially increase Chk2 ablation. On the molecular level our IHC data are consistent with published data showing phosphorylation by Chk2 at sites in the N- and C-terminal domains of p53 is required for fulminant stimulation of transcription from p53-target genes [12].

Considering that myelosuppression is one of the most frequent occurring DLTs seen in patients subjected to chemotherapy, we aimed to further investigate if targeting of Chk2 could prevent this type of toxicity in mice. Bone marrow (BM) aspirates from wild type and Chk2 (Chk2-/−) mice subjected to WBR were assessed for the presence of apoptotic cells with sub-G_1_ DNA content by flow cytometry. Indeed, BM aspirates from irradiated wild type mice showed a higher percentage of nucleated cells with sub-G_1_ content compared to mice lacking Chk2 (Figure 1B). Our interpretation of this finding is that Chk2 is a mediator of apoptosis also in the BM and this may contribute to Chk2-dependent myelosuppression following DNA damage inflicted by ionizing radiation.

### Chk2 triggers apoptosis in normal cells following select chemotherapeutics

In order to facilitate *in vitro* screening of chemotherapeutic contexts where inhibition of Chk2 may be most beneficial to prevent DLT's, we generated non-malignant E1A-immortalized MEF's from wild type (WTE1A) and Chk2-/− (Chk2-/−E1A) mice. In contrast to normal MEF's, which undergo senescence following DNA damage, E1A-transfected MEF's readily undergoes p53-dependent apoptosis following such cellular stress [13, 14]. We hypothesized that Chk2 may preferentially trigger cell death following DNA-damaging chemotherapeutics with certain genotoxic modes of action. Previous data have not addressed this facet of Chk2-targeting in detail. Subsequently we decided to undertake a small screen to identify chemotherapy that triggered cell death predominantly in a Chk2-dependent manner. Indeed, data from this screen indicated that the TOP2-inhibitors etoposide and doxorubicin triggered apoptosis in a Chk2-dependent manner (Figure 1C). In contrast, the TOP1-inhibitor CPT-11, the antimicrotubule agent taxol and the antimetabolite fluorouracil (5-FU) did not trigger cell death in E1A-immortalized MEF's in a Chk2-depedent manner (Figure 1C and data not shown). Interestingly, the proteasome inhibitor MG132 triggered apoptosis in the MEF's in a Chk2-dependent manner. Previous data have shown that MG132 can force accumulation of nuclear p53 potentially indicating that cell death was p53- and Chk2-dependent following inhibition of proteasomal degradation. Consistent with data from our screen, immunocytochemistry indicated that WTE1A MEF's expressed higher levels of p53, cleaved caspase-8 and more readily underwent apoptosis compared to Chk2-/−E1A MEF's following treatment with the TOP2-inhibitor etoposide (Figure 1D). Western blot assessment of PARP cleavage and cleavage of caspase-9 (CC9) showed that Chk2-deficient MEF's were increasingly protected from PARP and caspase-9 cleavage following treatment with etoposide compared to MEF's with intact Chk2 (Figure 1E). The ratio of cleaved PARP (p89) to full-length PARP (p116) ratio (p89:p116) and the normalized band density of CC9 for the highest dose of etoposide was 1.25 and 2.27 respectively for WT MEF's compared to 0.37 and 0.32 respectively for Chk2-/− MEFs. This indicates that induction of etoposide-induced apoptosis is deficient following loss of Chk2. In comparison following treatment with the TOP1-poison CPT-11, only limited expression of PARP p89 and CC9 was observed indicating modest onset of apoptosis downstream and canonical ATM-Chk2-p53 signaling following CPT-11. Moreover, little relative protection was observed from Chk2-deficiency with respect to the expression of cleaved PARP (the p89:p116 ratio was 0.09 and 0.07 respectively for wild type and Chk2 “null” cells respectively following 1.6 μM of CPT-11) and CC9 (the normalized CC9 band density of 0.83 and 0.61 was observed for WTE1A and Chk2-/−E1A MEF's respectively following 1.6 μM of CPT-11) (Figure 1E). To some extent our observations are consistent with previous studies where Chk2 was found to be a facilitator of chemotherapy- and IR-induced apoptosis in MEF's and normal mouse hematopoietic tissues [11, 15]. However, our data indicates that not all DNA damaging chemotherapy triggers apoptosis and toxicity in a Chk2-dependent manner.

**Figure 2:**
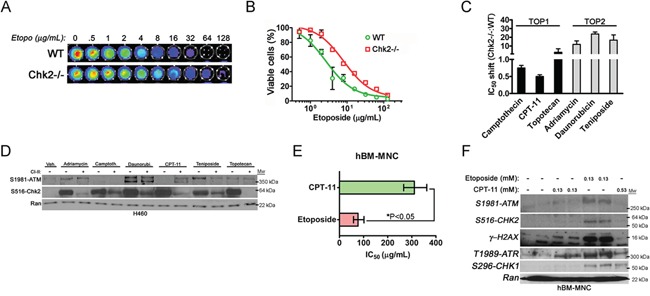
Chk2 is a mediator of toxicity triggered by TOP2-poisons **A.** The viability of primary mouse splenocytes isolated from wild type (WT) and Chk2-/− mice following treatment with etoposide *in vitro* was assessed by the CellTiter-Glo^®^assay. **B.** The dose-response IC_50_ for primary WT and Chk2-/− mouse splenocytes following long-term (72-hrs) treatment with etoposide was determined by the CellTiter-Glo^®^ assay. Error bars represent the standard error from the mean. N=3/treatment and genotype. **C.** The IC_50_-shift was determined for TOP1- and TOP2-inhibitors in primary splenocytes isolated from littermate Chk2-/− and WT mice. Error bars represent the standard error from the mean. N=3/treatment and genotype. **D.** Protein expression as detected by western blotting of phosphorylated ATM and Chk2 at their autophosphorylation sites S1981 and S516 respectively following 6 hours of treatment of the human lung cancer cell line H460 with TOP1 and TOP2-inhibitors. **E.** The IC_50_'s (μg/mL) for primary human bone marrow mono nucleated cells (hBM-MNC) treated with the TOP1- and TOP2-inhibitors CPT-11 (CPT-11) and etoposide for 24-hrs respectively were determined using the CellTiter-Glo® system. The error bars represent the standard error from the shown mean. *P<0.05, Student's t-test. **F.** Western blot of lysates from hBM-MNC's treated with isomolar (0.13 mM) and isotoxic (IC_50_) doses (0.13 mM and 0.53 mM) of etoposide and CPT-11 for 6-hrs respectively. The expressions of ATM, Chk2, ATR and Chk1 phosphorylated at the indicated autophosphorylation sites were assessed. Gamma-(γ) H2AX is a downstream substrate for the ATM kinase and marker for the presence of DSB. Ran was used as a loading control.

We also assessed Chk2-dependent killing of primary splenocytes isolated from wild type (WT) and Chk2 “null” (Chk2-/−) mice following treatment with etoposide (Figure 2A). The dose-response analysis indicated that Chk2-/− splenocytes displayed an approximately 3-fold higher IC_50_ *in vitro* compared to WT splenocytes following etoposide-treatment (10.18 [95%CI: 8.651-11.97] vs. 3.274 μg/ml [95% CI: 2.522 - 4.250]) suggesting protection from Chk2-deficiency over a broad dose-range of etoposide (Figure 2A, 2B and Table 1). In conclusion our data indicates that Chk2 may trigger toxicity in normal cells following some DNA damaging chemotherapy but not others.

**Table 1:** IC_50_ values to etoposide of wild type and Chk2-deficient splenocytes

Splenocytes	IC_50_ (μg/mL)	95%-CI	IC_50_ ratio	95%-CI
***Wild-type***	3.274	2.522 - 4.250	-	-
***Chek2^−/−^***	10.18	8.651 - 11.97	3.039	2.202 - 3.876

### Chk2 trigger cell death following treatment with TOP2-inhibitors but not following inhibitors of TOP1

TOP1-inhibitors trigger predominantly DNA single strand breaks (SSB) throughout the cell cycle that are converted to DSBs during S-phase when escaping cell cycle checkpoints and subsequent DNA repair. In contrast, TOP2-inhibitors have the capacity to trigger DSB's in all phases of the cell cycle that are highly lethal in G2/M phase. In order, to address the generality of our finding we assessed the dose-response parameters of a panel of TOP1- and TOP2-inhibitors in a primary mouse splenocytes isolated from wild type and Chk2-deficient mice. Indeed, an IC_50_-shift was observed in Chk2-deficient splenocytes predominantly following treatment with TOP2-inhibitors (Figure 2C). In contrast a limited IC_50_-shift (or even sensitization in the case of CPT-11) was observed following treatment with TOP1-inhibitors. This gave further support to the hypothesis that Chk2 controls toxicity following certain types of DNA damage such as that inflicted by TOP2-poisons.

It has previously been shown that DSB is the predominant activator the canonical ATM-Chk2-p53 pathway but Chk2 may also be activated by ATR and SSB's as a result of e.g. collapsed replications forks during S-phase. In support of this, western blot analysis of the p53-proficient and exponentially growing H460 lung cancer cell line treated with the panel of TOP1- and TOP2-inhibitors was performed. It has previously been shown that this cell line elicit a robust DDR including phosphorylation of Chk-proteins following DNA damage [16, 17]. Indeed, this analysis showed increased ATM-activation as evident by phosphorylation at the ATM autophosphorylation site S1981 following treatment with TOP2-poisons (Figure 2D). However, Chk2-activation, as evident by expression of S516 phosphorylated Chk2, occurred following treatment with both TOP1 and TOP2-poisons and did not correlate well with ATM-activation (Figure 2D). Furthermore, pharmacologic inhibition of Chk2 using Chk2 inhibitor II (CI2) suggested that in the case of CPT-11, Chk2 targeting might result in increased ATM activation perhaps as a result of increased DNA damage. Thus in rapidly dividing cancer cells Chk2 may become activated in an ATM-independent manner.

We also addressed activation of the ATM-Chk2 and ATR-Chk1 signaling following treatment with equimolar and equitoxic doses of CPT-11 and etoposide in normal primary human bone marrow mono nucleated cells (hBM-MNC). Human BM-MNC's show limited proliferation in culture where more than ninety-five (95) percent of the cells remain in G_0_-G_1_ phase of the cell cycle (data not shown). We first determined the BM-MNC's IC_50_ to CPT-11 and etoposide and then subjected them to short-term (6 hrs) treatment to equitoxic doses of the TOP1- and TOP2-poison respectively that did not trigger cell death (Figure 2E and 2F). Indeed, western blot data indicated that etoposide more robustly activated ATM-Chk2 signaling compared to CPT-11 (Figure 2F). Moreover, at equitoxic doses (0.13 mM) etoposide was more potent in the triggering of DSB as evident of an increased expression of gamma-H2AX following such treatment (Figure 2F). However, CPT-11 was in comparison to its relative potency to trigger expressions of phosphorylated species of ATM and Chk2 increasingly potent in activating ATR at both isomolar (0.13 mM) and equitoxic doses (0.53 mM) and Chk1 S296 autophosphorylation at equitoxic doses. Thus this finding suggests that etoposide increasingly triggers a DDR that activates ATM and Chk2 in the bone marrow whereas the DDR following CPT-11 involves a more isolated activation of ATR and Chk1 *in vitro*.

### Chk2 controls toxicity in mice *in vivo* following the TOP2-poison etoposide

**Figure 3:**
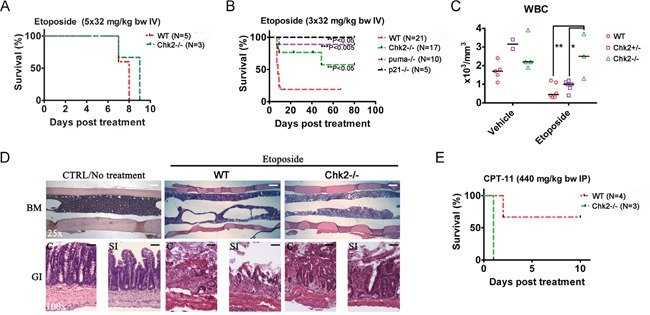
Chk2 triggers dose-limiting toxicity in mice *in vivo* following etoposide **A.** Wild type and Chk2-/− mice were treated with etoposide once daily for five (5) days at the indicated doses. **B.** Wild type, Chk2-/− and mice lacking the p53 responsive apoptosis- and cell cycle regulating genes puma (puma-/−) and p21 (p21-/−) were treated every other day for 5 days with etoposide. Statistical significant differences (P<0.05) in survival relative to treated wild type mice were analyzed by log rank (Mantel Cox) tests. **C.** Hematology assessment in wild type (WT), Chk2+/− and Chk2-/− mice suggest a Chk2-dependent reduction in the white blood cell count (WBC) following etoposide. Each data point represents data from one mouse. Black lines indicate the median of each group. “*” Indicates a P<0.05 and “**”indicate a P<0.01 by 1-way ANOVA analysis with Bonferonni correction. **D.** Histology (H/E staining) of the bone marrow (BM), colon (C) and small intestine (SI) of mice succumbing to etoposide-treatment. Size bars represent 100 μm (25x) and 25 μm (100x). **E.** Survival of wild type and Chk2-/− mice treated with EC_50_-doses of CPT-11.

In order to assess the importance of Chk2 in mediating DLT's following chemotherapy, we employed an *in vivo* mouse model for treatment with the TOP2 poison etoposide. This model was based on a clinical repeat-dose etoposide-treatment protocol, where etoposide is administered three (3) to five (5) times during a period of five days. Surface area doses of etoposide employed in patients were converted to doses for IV injection in mice according to Freireich et al. [18]. Interestingly, when etoposide was injected five times during the time course of 5 days (5×32 mg/kg bw IV), no significant contribution of Chk2 to toxicity *in vivo* was observed since both wild type and mice lacking the Chk2 gene succumbed following this treatment schedule (Figure 3A). However, when the accumulated dose of etoposide was reduced to 96 mg/kg (3x32 mg/kg bw IV) and given over 5 days with one day in between, a significant protection was observed in mice lacking Chk2 as well as in mice lacking the p53-responsive genes p21 (p21-/−; a cell cycle inhibitory protein) and Puma (puma-/−; a pro-apoptotic BH3-only protein) (Figure 3B). Furthermore, wild type and mice heterozygous for Chk2 showed reduced levels of circulating white blood cells (WBC) following treatment with two doses of etoposide (2x32 mg/kg bw) compared to mice that completely null for Chk2 (Figure 3C). As expected, toxicity in this model was associated with BM and gastrointestinal aplasia (Figure 3D). Consistent with the previous *in vitro* findings, when a toxic (EC_50_) dose of the TOP1-poison CPT-11 was administered to Chk2-/− mice, no apparent protection was observed (Figure 3E). In contrast, such mice appeared to be sensitized when compared to wild type mice. Thus our data are consistent with the notion that Chk2 trigger DLT following TOP2-poisons. In contrast, Chk2 may not trigger toxicity following TOP1-poisons *in vivo*.

### Pharmacologic inhibition of Chk2 prevents killing of normal cells *in vitro* by the TOP2-poison etoposide

**Figure 4:**
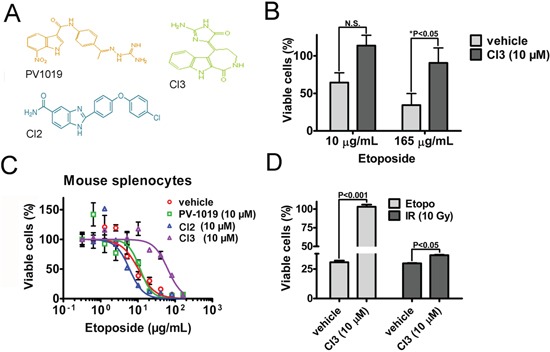
Pharmacologic inhibitors of Chk2 (Chk2i) protects normal human and mouse cells from etoposide-induced killing **A.** The chemical structures of PV1019, CI2 and CI3 **B.** Quantitation of MRC5 cell survival following etoposide-treatment in the presence and absence (vehicle) of CI3. The statistical significance was determined by the two-way ANOVA test with Bonferroni multiple comparison correction. The relevant ‘P’ values are indicated for N=6. **C.** Twenty-four (24) hour dose-response CellTiter-Glo® assay assessing the survival of primary mouse splenocytes isolated from C57BL6J mice in the presence and absence of Chk2i following treatment with etoposide. The curve fit was analyzed using the GraphPad Prism 5.0 software. IC_50_'s with non-overlapping 95%-confidence intervals (CI) were considered statistically significantly different. An N=3/treatment was employed for this particular experiment. **D.** The cell viability of mouse splenocytes relative untreated control was determined by the CellTiter-Glo® assay. The assay was performed at twenty-two (22) hours following treatment with etoposide (18 μg/mL) and 10 Gy of ionizing radiation (IR) in the presence and absence of the Chk2i CI3. The splenocytes were pre-treated with CI3 two (2) hours prior to initiation of treatments. Error bars represent the standard error from the mean. Statistical assessment was performed by a two-way ANOVA test with Bonferroni multiple comparison correction.

In order to verify if our findings with regard to genetic ablation of Chk2 translates to pharmacologic (Chk2i) targeting of Chk2's kinase function we treated human normal fibroblasts with etoposide and a panel of Chk2i's. Our data indicates that CI3 [9] protected the MCR5 cells from the toxic effect of etoposide following treatment with 165 μg/ml of the chemotherapeutic (Figure 4A and 4B). To address if the pharmacologic inhibitors would phenocopy splenocytes lacking the Chk2 gene we subjected wild type splenocytes to etoposide-treatment in the absence and presence of PV1019 [19], CI2 [7] and CI3 (Figure 4A and 4C). Our finding indicates that CI3 (IC_50_: 63.47 μg/mL, 95%CI: 51.63-78.03) offered mouse splenocytes a 6-fold protection from etoposide-induced toxicity compared to vehicle (IC_50_: 10.29 μg/mL, 95%CI: 7.484-14.15) (Table 2). We also subjected mouse splenocytes to ionizing radiation (IR) in the absence and presence of CI3 *in vitro* (Figure 4D). Here we observed a modest but statistically significant protection by CI3 from IR-induced killing of the splenocytes compared to vehicle (control) (36.7 ± 0.53 vs 29.8 ± 0.62 % respectively). In contrast, CI3 completely protected the splenocytes from etoposide-induced killing. From this we conclude that pharmacologic targeting of Chk2 by Chk2i such as CI3 may be particularly potent in protecting from cell death from TOP2-poisons such as etoposide. Thus it may be a feasible approach to pharmacologically inhibit Chk2 to countermeasure DLT's following etoposide treatment.

**Table 2:** Splenocyte IC_50_ to etoposide without and with Chk2i

Splenocytes	IC_50_ (μg/mL)	95%-CI	IC_50_ ratio	95%-CI
***Vehicle***	10.29	7.484 – 14.15	-	-
***PV-1019 (10 μM)***	11.52	8.996 – 14.76	1.275	0.8781 – 1.673
***CI2 (10 μM)***	5.952	5.149 – 6.881	0.6690	0.4605 – 0.8776
***CI3 (10 μM)***	63.47	51.63 – 78.03	6.949	4.773 – 9.126

### A combined in silico and functional compound screen identifies NSC105171 as a Chk2i with toxicity countermeasure activity

**Figure 5:**
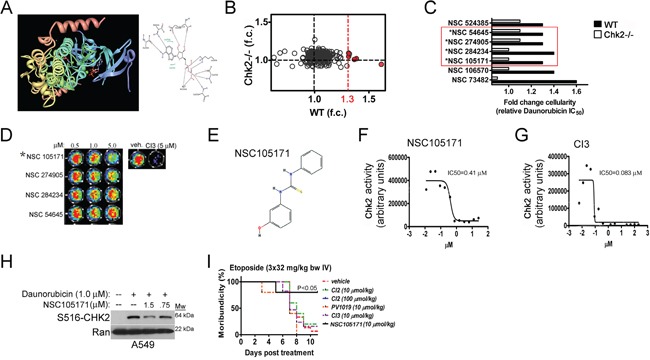
A combined in silico and genetic screen identifies NCS105171 - a novel lead Chk2i with countermeasure activity *in vitro* and *in vivo* **A.** Construction of in silico docking protocols using the high-resolution crystal structure of ADP bound to the Chk2 kinase. **B.** Identified lead compounds from the condensed compound library that protects mouse primary splenocytes from cell death following treatment with the TOP2-poison daunorubicin. The cutoff was set to 1.3-fold (indicated in red) for protection. **C.** Four lead compounds that show Chk2-dependent protection from daunorubicin-dependent cell death. **D.** The kinase inhibitory activity of the lead Chk2i's was assessed in a cell-free Chk2 kinase assay. **E.** The chemical structure of the lead compound NSC105171 (ptu-23) is depicted. Graphs showing dose-dependent inhibition of Chk2 kinase activity by NSC105171 **F.** and CI3 **G.** in the Chk2 kinase assay. **H.** NSC105171 inhibits the expression of Chk2 phosphorylated at the autophosphorylation site Serine 516 in human A549 lung cancer cells following treatment with daunorubicin. **I.** Survival of mice subjected to repeat-dose exposure of etoposide and co-treated with commercially available Chk2i's and NSC105171.

Although the Chk2i's showed promising activity following treatment with etoposide *in vitro*, they failed to prevent toxicity following etoposide-treatment *in vivo* (Figure 5I). Unfortunately, delivering the Chk2i's by surgically implanted osmotic pumps in mice did not improve activity in this model and it also became apparent that some of these Chk2i's triggered increased weight loss in the etoposide-treated mice (data not shown). Therefore, in order to identify lead Chk2i with *in vivo* countermeasure activity we designed a combined computational, functional and cell-based screen for such compounds (Figure S1). The chemical structures present in the Diversity Set II (NCI/NTP) were compared to the crystal structure of ADP bound to Chk2 from the Protein Data Base (PDB: 2CN5). From this approach we were able to condense the number of candidate compound structures from 1,364 to 299 (22% of the initial library size) (Figure 5A). Subsequently, we used this condensed compound library to screen wild type (WT) and Chk2-/− mouse splenocytes for protection (improved cell survival) to isotoxic doses (defined from genotype-specific IC_50_'s) of the TOP2-poison daunorubicin (Figure 5B and 5C). The inclusion of cells devoid of Chk2 was performed in order to omit compounds that offered protection in a manner independent of Chk2. Furthermore, in order to trigger potent Chk2-activation in the splenocytes we used the TOP2-poison daunorubicin. Our data indicate that daunorubicin was most potent at killing splenocytes through Chk2 since splenocytes lacking the Chk2 gene had an approximately 30-fold higher IC_50_ compared to that of wild type splenocytes (Figure 2C and 2D). The Z-scores for the functional screens of the wild type and Chk2-/− splenocytes were 0.89 and 0.87 respectively indicating ‘excellent’ sensitivity in both assays [20]. Based on the results from the combined screens we chose a cut-off for protection at 1.3-fold change (f.c.) (Figure 5B). Furthermore, we also assessed the compounds of the condensed library for inhibition of Chk2 activity in a cell-free kinase assay (Figure 5D and Figure S1).

A total of seven (7) compounds were found to improve the survival of wild type but not Chk2-/− splenocytes following treatment with daunorubicin (Figure 5C). Three (3) of these compounds were removed from further assessment based on information retrieved from e.g. the TOXLINE database (http://toxnet.nlm.nih.gov/) indicating unfavorable toxicities. For example, the caffeine derivative NSC524385 was somewhat less potent than the other lead compounds. Furthermore, caffeine is a potent inhibitor of ATM, a kinase upstream of Chk2 required for fulminant Chk2 activity following DSB's and triggers toxicity in laboratory animals. NSC106570 is a muscle relaxant and a psychotropic drug which makes this lead compound less suitable for further *in vivo* assessment.

### NSC105171 is a pharmacologic countermeasure to etoposide-induced toxicity *in vivo*

The four (4) remaining compounds (red box) were re-evaluated for inhibition Chk2-activity in the kinase assay (Figure 5C and 5D). Only NSC105171 (E) were found to sufficiently inhibit CHK2-dependent phosphorylation of a synthetic oligopeptide encoding for a Chk1/2 -phospho site of CDC25A, in the presence of recombinant Chk2 and ATP (Figure 5E–5G). NSC105171 showed an IC_50_ that was approximately eight (8) times higher than the hymenialdesine derivative CI3 [9] (Figure 5F and 5G). This indicates that in comparison, NSC105171 is a less potent Chk2i *in vitro*. In A549 cells, NSC105171 inhibited the expression of Chk2 phosphorylated at the autophosphorylation site Serine 516 (S516) following treatment with daunorubicin indicating cell *in vivo* activity (Figure 5H). We decided to test the efficacy of NSC105171 in preventing etoposide-induced toxicity in mice *in vivo*. As previously noted, neither of the three Chk2i's that show activity in cells *in vitro* (CI2, CI3 and PV1019) protected mice from etoposide-induced toxicity compared to mice subjected to vehicle (Figure 5I). In contrast, NSC105171 did provide significant protection (P<0.05; log rank test, N=5/treatment group) compared to vehicle-treated mice and this finding was correlated improved bone marrow histology (Figure 5I and ‘data not shown’). This indicates that the combined *in silico* and functional screening approach may identify lead Chk2i for *in vivo* application to countermeasure DLT's from TOP2-poisons.

## DISCUSSION

Inhibition of Chk2 may offer a novel strategy of clinical intervention to prevent DLT's from DNA damaging chemotherapy. Inhibition of Chk2 may curtail propagation of DDR signals that trigger hematopoietic stem cell death and BM aplasia. This molecularly targeted strategy takes advantage of a common differences between normal and cancer cells in their capacity to trigger p53-dependent cell death following DNA-damaging therapies since cancer cells frequently carry inactivating p53-mutations [21]. Moreover, some cancers frequently show high expression of T68 phosphorylated Chk2 indicating high activity of Chk2. A genomic and proteomic study of the NCI-60 cell line panel indicated that 12% of the panel had high endogenous activation of Chk2 and this was always associated with loss of p53 [22]. It has previously been shown that cancer cells lacking functional p53 may be increasingly sensitive to damage that trigger G2/M checkpoints such as that by anti-mitotics [23, 24]. Indeed, Chk2 may stimulate DNA repair and exercise increased control of cell cycle progression through G_2_/M transition in the absence of p53, something that may diminish the potency of DNA damaging therapy in some cancer cells. Subsequently, Chk2 inhibition may both sensitize cancer cells to DNA damage and at the same time prevent DLT's.

Our data indicate that Chk2 induces cell death following DNA damage in a highly chemotherapeutic-specific manner. For example, targeting of Chk2 may have limited value as a toxicity countermeasure following treatment with TOP1-inhibitors since loss of Chk2 did not protect E1A-immortalized MEF's from cell death following treatment with CPT-11 nor did somatic loss of the Chk2 gene protect mice from lethal toxicity of CPT-11. Furthermore, loss of Chk2 did not change the dose-response relationship of splenocytes to a panel of TOP1-inhibitors *in vitro* significantly. These observations stand in contrast to data on a panel of TOP2-inhibitors where loss of Chk2 in mouse splenocytes caused a profound protective increase of the IC_50_'s. Moreover, *in vivo* data indicate that mice devoid of Chk2 are protected from lethal toxicities of the TOP2-inhibitor etoposide. Based on this we conclude that pharmacologic inhibition of Chk2 to prevent DLT's may be most efficient following chemotherapy such as TOP2-inhibitors.

In contrast to TOP1-inhibitors, TOP2-inhibitors trigger DSB directly through inhibition of TOP2alpha and may rely increasingly on canonical ATM-Chk2-p53 signaling pathway throughout the cell cycle [25]. Inhibition of TOP1 may trigger toxicity primarily through the generation of SSB and the generation of pathological levels of single stranded DNA (ssDNA) following replication fork stalling. As a consequence of this, this type of cellular DDR may rely increasingly on the activation of ATR and Chk1 and less on activation of ATM and Chk2 [26, 27]. This would be particularly apparent in organs such as the BM, that under unstressed conditions display a low frequency of cycling cells. In such organs, SSB's inflicted by TOP1-inhibitors may be slowly and infrequently converted to DSB's [28]. This is consistent with our data on primary human BM cells that indicate limited activation of ATM and Chk2 following treatment with CPT-11 compared to treatment with equitoxic doses of etoposide. In the absence of Chk2, cells might be less prone to instigate p53-dependent G_1_-S and intra-S checkpoints [29]. Subsequently, loss of Chk2 may on the contrary render normal tissues with frequently cycling cells such as the GI tract susceptible to TOP1-inhibitors that rely on S-phase progression to generate lethal DSB's.

In order to isolate novel Chk2i suitable as countermeasures to DLT's of TOP2-poison, we undertook a combined in silico and functional compound screen. Our collective efforts suggested that the carbanilide-derivate NSC105171 inhibit the kinase activity of Chk2 and protect Chk2-proficient mouse splenocytes from daunorubicin-dependent killing. Interestingly, NSC105171 is also known as the carbanilide derivative ptu-23, an antiviral compound that protect mice from lethal infections of several Coxsackie virus strains [30, 31]. Furthermore, NSC105171 appears to have a permissive safety profile with a reported mouse LD_50_ of 1,000 mg/kg bw. The combination of a permissive toxicity profile and *in vivo* activity of NSC105171 has spurred us to investigate NSC105171-analogues as a novel class of small molecule *in vivo* competent Chk2i (to be published elsewhere).

In summary, our data indicates that Chk2 is the predominant trigger of toxicity following TOP2-inhibitors and incorporating Chk2i's into chemotherapy protocols that employ TOP2-inhibitors may fully take advantage of the benefits such strategy's have to offer for clinical translation.

## MATERIALS AND METHODS

### Mice and treatments

Six to eight-week old male C57BL/6J (wild type), C57BL/6-Bbc3tm1Ast/J (puma-/−) and B6.129S6 (Cg)-Cdkn1atm1Led/J (p21-/−) were purchased from Jackson Laboratory (Jackson Laboratory, ME). Chk2-/− mice have been described previously [11]. All mice were housed in a controlled environment with regard to light, temperature and humidity. An Institutional Animal Care and Use Committee approved all animal care and treatment procedures employed.

### Cell culture, cell viability assays, and reagents

For cell viability assays, cells were seeded into 96-well black-walled plates at a concentration of 2×10^5^ cells (splenocytes) per well in fresh media and in a volume of 100 μL per well. At endpoint, CellTiter-Glo® (Promega) assays were performed according to the manufacturer's protocol.

### Flow cytometry

Single cell suspensions were prepared from the tibia and femur and analyzed for sub-G_1_ FACS analysis. Bone marrow cells were collected and fixed with 70% ethanol at 4°C. The samples were stained with propidium iodide (Sigma) in the presence of RNase and subjected to flow cytometric analysis using Epics Elite flow cytometer (Beckman Coulter, Fullerton, CA).

### Retroviral transfection

The generation of E1A-immortalized MEF's was performed in a similar manner as described previously [32].

### Statistical analysis

The statistical significance of differences between data sets was analyzed by either Two-Way ANOVA with Bonferonni correction or the log-rank (Mantel-Cox) test using the GraphPad Prism software. P<0.05 was considered as statistically significant.

### In silico screening of compound libraries

The crystal structure of human CHK2 in complex with ADP, debromohymnialdisine-derived inhibitors and NSC 109555 was retrieved from the Protein Data Bank (PDB: 2CN5, 2CN08, 2W07 [33, 34]). To generate an optimally performing docking protocol, ADP and NSC 109555 were re-docked to the ADP-binding pocket of the crystal structure of human CHK2 (PDB: 2CN5, 2CN08, 2W07) with several combinations of scoring and algorithms for docking function using the Schrödinger Small-molecule Drug Discovery Suite. This docking protocol was subsequently applied to the Diversity Set II compound library (DTP/NIH).

### Histology and immunohistochemistry

Following necropsy, the bone marrow, colon, small intestine, spleen, testis and thymus were collected and fixed in 4% paraformaldehyde overnight at 4°C, embedded in paraffin and cut into 4-μm sections for evaluation by histology and immunohistochemistry. Cut sections were stained as previously described [32, 35].

### Western blotting

Western blotting was performed as described previously [32, 36]. Densiometry on bands was made using the NIH Image J 1.45S software.

### Statistical analysis

The statistical significance of differences between data sets was analyzed by either Two-Way ANOVA with Bonferroni correction or the log-rank (Mantel-Cox) test using the GraphPad Prism software. P<0.05 was considered as statistically significant.

## SUPPLEMENTARY FIGURE



## Abbreviations

ATM: Ataxia telangiectasia mutated
BM: bone marrow
Chk2: cell cycle checkpoint kinase 2
Chk2i: Chk2 inhibitors
CI2: cell cycle checkpoint inhibitor II
CI3: cell cycle checkpoint inhibitor III
DDR: DNA damage response
DLT: dose-limiting toxicity
DSB: DNA double-strand break
IR: γ-radiation
S516: Serine 516
MEF: mouse embryo fibroblasts
SSB: DNA single-strand break
ssDNA: single-stranded DNA
TOP1: topoisomerase-I
TOP2: topoisomerase-II
T68: Threonine 68
WBR: Whole Body Irradiation
