# Supplementary material for: Targeting of Chk2 as a countermeasure to dose-limiting toxicity triggered by topoisomerase-II (TOP2) poisons
Source: Oncotarget. 2016 Apr 18;7(20):29520–30. doi: 10.18632/oncotarget.8790 (PMC5045414; doi:10.18632/oncotarget.8790)
Supplement: Supplementary file 1 [file oncotarget-07-29520-s001.pdf]

SUPPLEMENTARY INFORMATION

MATERIALS AND METHODS

Mice and treatments

Six to eight-week old male C57BL/6J (wild type), C57BL/6-Bbc3tm1Ast/J (puma-/-) and B6.129S6 (Cg)-Cdkn1atm1Led/J (p21-/-) were purchased from Jackson Laboratory (Jackson Laboratory, ME). Chk2-/- mice have been described previously (9). All mice were housed in a controlled environment with regard to light, temperature and humidity. Mice were euthanized through cervical dislocation following ketamine/xylazine anesthesia. An Institutional Animal Care and Use Committee approved all animal care and treatment procedures employed. Four- to six-week-old wildtype and Chk2−/− mice were subject to 5 Gy of whole-body radiation (WBR) with a ^{137}Cs gamma source at a rate of 1.4 Gy/min. At 6 hrs following radiation animals were euthanized with an approved Institutional Animal Care and Use Committee Protocol, which followed the recommendations of the Panel on Euthanasia of the American Veterinary Medical Association. The femur and tibia of the animals were isolated and aspirated with RPMI-1640 medium supplemented with FBS and penicillin and streptomycin. Red blood cells were lysed in ACK buffer (0.15 M NH_4Cl, 10.0 mM $KHCO_3$, 0.1 mM Na_2EDTA, pH 7.4) and the remaining bone marrow cells were washed and resuspended in cold PBS. The bone marrow cells were then stained with propidium iodide and subjected to flow cytometric sorting to quantitate the percentage of cells with sub-G_1 DNA (see below). For the *in vivo* etoposide-toxicity model, mice were injected with etoposide at a dose level and protocol equivalent to a clinical relevant protocol of etoposide treatment. Subsequently etoposide was injected IV at 32 mg/kg bw three times over 5 days with one day of rest between doses. The body weight of the mice was measured throughout the study. For hematology of etoposide-treated mice, wildtype and Chk2−/− animals were subjected to two (2) consecutive doses of etoposide (32 mg/kg bw IV). Mice were euthanized two days following the last dose of etoposide and blood was collected by cardiac puncture and submitted for analysis.

Cell culture, cell viability assays, and reagents

Cell lines were obtained from ATCC and cultured in ATCC-recommended media in a humidified incubator at 5% CO2 and 37°C. For cell viability assays, cells were seeded into 96-well black-walled plates at a concentration of 2×10^5 cells (splenocytes) and 50,000 cells (cancer cell lines) per well in fresh media and in a volume of 100 µL per well. Cells were allowed to adhere overnight and were treated the next day as indicated. At endpoint, CellTiter-Glo™ (Promega) assays were performed according to the manufacturer's protocol, and the bioluminescent readout was recorded on an IVIS imaging system (Xenogen). Camptothecin, teniposide, etoposide, daunorubicin, adriamycin, topotecan, taxol and CPT-11 was obtained from Penn State Hershey Cancer Institute Pharmacy. MG132 was purchased from Sigma-Aldrich. Chk2 inhibitors PV1019 (6), CI2 (5) and CI3 (7) were purchased from EMD Millipore. CellTiter-Glo™ was purchased from Promega.

Splenocyte isolation and compound screening

Splenocytes were isolated from 4 – 6-week old wildtype and Chk2-/- mice and cultured in 96-well plates at 1.0 x 10^5 cells/well in RPMI 1640 medium supplemented with 10% fetal bovine serum (Hyclone) and antibiotics. The splenocytes were subjected to treatment for twenty-four (24) hours with different chemotherapy after which cell viability was assessed by using CellTiter-Glo® (Promega) and a xenogen imaging system. For the determination of compound protection from Chk2-dependent killing wildtype and Chk2-/- splenocytes were treated with equitoxic (IC_{50}) doses of daunorubicin for twenty-four hours in the presence and absence of 5 µM of compounds from the in silico condensed compound library and the cell viability was determined using the CellTiter-Glo® assay.

In silico screening of compound libraries

The crystal structure of human CHK2 in complex with ADP, debromohymnialdisine-derived inhibitors and NSC109555 was retrieved from the Protein Data Bank (PDB: 2CN5, 2CN08, 2W07 (38,39)). The electrostatic Potential (ESP) methodology was used to calculate charges (with the help of the R.E.D. Server (40)) to help assess inter-molecular dynamic interactions within the ADP-binding pocket of Chk2. Docking protocols were generated using MOE-Dock (http://www.chemcomp.com) GOLD (www.ccdc.cam.ac.uk/) and Glide (41). To generate an optimally performing docking protocol, ADP and NSC109555 were re-docked to the ADP-binding pocket of the crystal structure of human Chk2 with several combinations of scoring and algorithms for docking function using the Schrödinger Small-molecule Drug Discovery Suite. This docking protocol was subsequently applied to the Diversity Set II compound library (DTP/NIH).

Flow cytometry

Single cell suspensions were prepared from the tibia and femur and analyzed for sub-G1 FACS analysis. Bone marrow cells were collected and fixed with 70% ethanol at 4°C. The samples were stained with propidium iodide (Sigma) in the presence of RNase and subjected to flow cytometric analysis using Epics Elite flow cytometer (Beckman Coulter, Fullerton, CA).

Retroviral transfection

The generation of stable p53 knock-down human colorectal cancer cell lines was performed in a similar manner as described previously for mammary epithelial cells (10). Human colorectal cancer cells HCT116 were grown as described previously (42). Briefly, amphotrophic retroviruses were made by transfecting Phoenix-Ampho cells with the Lipofectamine2000 reagent (Invitrogen) by following the manufacturer's instructions. After 2 days of transfection, the filtered viral supernatant was used to infect target HCT116 cells using spin centrifugation. Retroviruses were infected serially and cells having puromycin resistance (together with scrambled shRNA or shRNA targeting p53) were isolated and subsequently expanded *in vitro*. The generation of E1A-immortalized mouse embryo fibroblasts (MEF) was performed in a similar manner as described previously (11). Ecotropic Phoenix cells were transfected with LPC-E1A (kindly provided by Dr. Scott Lowe, Memorial Sloan-Kettering Cancer Center). The supernatant containing recombinant retrovirus was harvested 48 h following transfection. Wildtype and Chk2-/- MEF's were seeded at a density of 10^5 and incubated 1 h with the retroviral supernatant. Cells stably expressing E1A were selected by incubation with either 2 µg of puromycin per ml for 3 days.

Histology and immunohistochemistry

Following necropsy, the bone marrow, colon, small intestine, spleen, testis and thymus were collected and fixed in 4% paraformaldehyde overnight at 4°C, embedded in paraffin and cut into 4-µm sections for evaluation by histology and immunohistochemistry. Cut sections were stained with hematoxylin and eosin (H&E) and analyzed by microscopy. Immunohistochemistry was performed by rehydrating slides and subjecting them to antigen-retrieval through boiling in 1 mM citric acid buffer (pH 6.0). Endogenous peroxidases were blocked by submerging slides in 3% H_2O_2. Briefly, primary antibodies were left on sections overnight at 4°C. Sections were washed and primary antibodies were conjugated with either peroxidase conjugates using the ImmPRESS™-system (Vector laboratories) counterstaining was performed using hematoxylin. Representative depiction of histology and immunohistochemistry was made using IP lab software (BD Biosciences).

Western blotting

Cells were homogenized and sonicated in RIPA-buffer (1xPBS, 1% NP-40, 0.5% sodium deoxycholate, 0.1% SDS, 1mM PMSF, *Complete* protease inhibitor cocktail (Roche)). Protein concentrations were determined by the Bradford method (Bio-Rad) and proteins separated on sodium dodecylsulfate 12.5% polyacrylamide gels and transferred to polyvinylidene difluoride membranes. Labeling of the transferred proteins were performed using the following primary antibodies: phospho-ATM (Rockland Immunochemicals), Thr1989-ATR (Genetex), S296-Chk1 (Cell Signal), S516-Chk2 (Cell Signal), cleaved caspase-9 (Asp353) (Cell Signal), PARP (Cell Signal) and Ran (BD Transduction Laboratories). Membranes were incubated with horseradish peroxidase conjugated secondary antibodies (1:4,000) and detected by the ECL procedure (Amersham).

Statistical analysis

The statistical significance of differences between data sets was analyzed by either Two-Way ANOVA with Bonferroni correction or the log-rank (Mantel-Cox) test using the GraphPad Prism software. $P<0.05$ was considered as statistically significant.

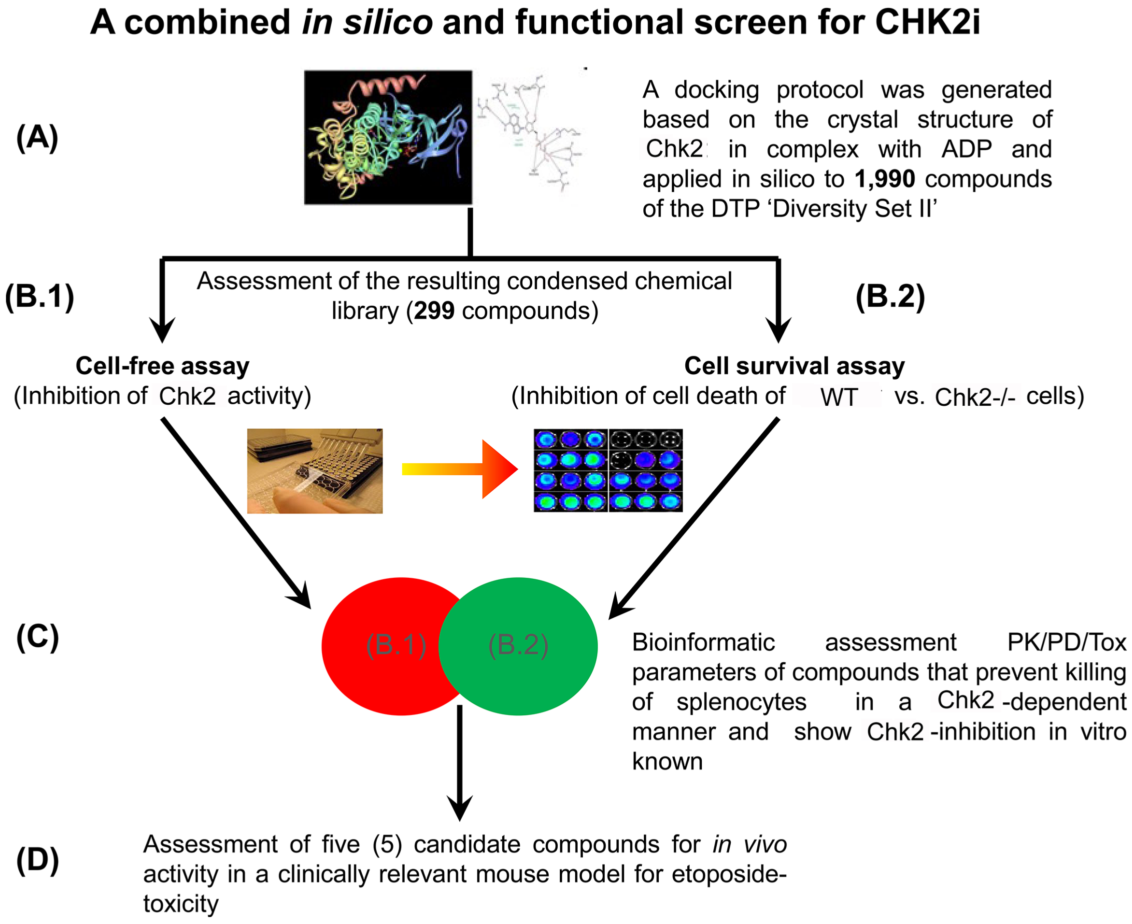

Supplementary Figure S1: Schematic showing the development of a screening strategy to isolate novel pharmacologic Chk2 inhibitors with *in vivo* activity. The approach shows an in silico docking protocol employed on the Diversity Set II (A) based on the high resolution crystal structure of ADP bound to Chk2. This was performed to help condense this compound library and allow for quicker identification of lead compounds in subsequent a cell-based screen. This functional screen utilized employed a cell-free kinase assay in parallel to assess the kinase inhibitory potency of the lead compounds (B.1). Furthermore, normal primary mouse splenocytes that with an intact (CHK2+/+) and mutated and functionally inactivated chek2 gene (CHK2-/-) (B.2) in order to help identify small molecule inhibitors of Chk2 (Chk2i) with minimal off-target effect. The candidates were assessed for favorable pharmacokinetics and toxicology data based on a priori knowledge through the use of the TOXNET data base (C) followed by further experimental assessment of activity (D).
